# From CAF subtypes to clinically relevant states: cancer-associated fibroblasts in pancreatic ductal adenocarcinoma

**DOI:** 10.3389/fimmu.2026.1971880

**Published:** 2026-10-06

**Authors:** Yi Hao, Yi Wang, Qinyang Du, Gaopeng Li

**Affiliations:** Third Hospital of Shanxi Medical University, Shanxi Bethune Hospital, Shanxi Academy of Medical Sciences, Tongji Shanxi Hospital, Taiyuan, China

**Keywords:** cancer-associated fibroblast, fibroblast state, pancreatic ductal adenocarcinoma, spatial transcriptomics, stromal heterogeneity, stromal reprogramming, therapy resistance, tumor microenvironment

## Abstract

Pancreatic ductal adenocarcinoma (PDAC) develops within a dense, poorly perfused tumor microenvironment (TME) in which cancer-associated fibroblasts (CAFs) are prominent cellular and structural components. Once viewed as a uniform population of activated matrix-producing cells, CAFs now include myofibroblastic, inflammatory, antigen-presenting and other populations identified by single-cell and spatial studies. These categories sharpen experimental resolution but do not form a stable hierarchy. CAF phenotypes overlap across spatial niches and disease stages, and they change under metabolic stress or treatment. We frame this heterogeneity as a set of context-dependent functional states. This review synthesizes evidence on fibroblast origins, canonical and recently reported states, and the signals that drive state transitions. It then examines CAF functions in extracellular matrix organization, tumor-cell plasticity and dissemination, metabolic support and immune regulation. Findings on tumor-restraining fibroblasts and protective collagen pools challenge indiscriminate stromal depletion. We also assess CAF states as prognostic and predictive biomarkers, their roles in treatment resistance and therapeutic strategies based on depletion, pathway blockade or stromal normalization. Failures of Hedgehog and hyaluronan-directed therapies show that target abundance alone cannot select patients. Clinical translation will require state definitions that integrate function, spatial context and treatment history, supported by reproducible markers, prospective validation and models that retain human stromal diversity.

## 1 Introduction

### 1.1 PDAC and its tumor microenvironment

Pancreatic ductal adenocarcinoma is marked by early dissemination, limited sensitivity to systemic treatment and frequent recurrence. Its tumor microenvironment (TME) differs from that of many other epithelial cancers because malignant glands often occupy a minority of the tissue. The remaining space contains extracellular matrix (ECM), fibroblasts, myeloid and lymphoid cells, vessels and nerves. These elements actively determine oxygen and nutrient availability, organize cell contact, carry paracrine signals and alter drug exposure. Treatment responses therefore emerge from a changing multicellular ecosystem (1).

Desmoplasia is the most visible feature of this ecosystem. Dense collagen and hyaluronan can raise interstitial pressure, compress vessels and create regions of hypoxia. These physical conditions coexist with cytokine and metabolic gradients. Human spatial analyses have shown that PDAC tissue contains recurrent regional microenvironments with different fibroblast programs, immune composition and effects on cancer cells (2, 3). Some regions are dominated by activated, inflammatory stroma, whereas others contain dense matrix and more differentiated myofibroblastic programs. Their coexistence within one tumor helps explain why a single biopsy or bulk expression score can miss clinically relevant variation.

Treatment also remodels the TME. Single-cell and spatial studies of human tumors exposed to chemotherapy, radiotherapy or immunotherapy have identified changes in malignant-cell state, fibroblast composition, myeloid recruitment and ligand-receptor interactions (3–6). Together, these observations describe continuous adaptation: cancer and nonmalignant cells respond to the same stress while modifying one another.

### 1.2 CAFs as regulators of PDAC biology

CAFs coordinate structural and signaling functions during this adaptation. They produce and remodel collagen, fibronectin, proteoglycans and basement-membrane proteins. They also secrete cytokines, chemokines, growth factors and metabolites and interact with cancer, immune, endothelial and neural cells. These activities affect tumor growth, invasion, immune surveillance and therapy response (7, 8). CAFs are local organizers of tissue function, not merely matrix-producing support cells.

This functional breadth complicates CAF identification. No single marker identifies all CAFs while excluding smooth-muscle cells, pericytes and other mesenchymal populations. Common markers such as α-smooth muscle actin (α-SMA), fibroblast activation protein (FAP), platelet-derived growth factor receptors and ECM genes identify overlapping subsets. Marker abundance also changes with activation and tissue handling. CAFs must therefore be defined operationally by combining morphology, location, multiple markers and, when possible, lineage or functional evidence (9, 10).

Opposing results from stromal targeting exposed the consequences of this uncertainty. Some experiments showed that CAF-derived factors or matrix components support cancer growth and resistance. In mouse PDAC, direct α-SMA-positive fibroblast depletion and fibroblast-derived collagen deletion each revealed tumor-restraining stromal effects (11, 12). Collectively, these studies assign distinct effects to different CAF states and ECM products. A target’s effect depends on the expressing cells, their location and the compensatory response to inhibition.

### 1.3 From traditional classification to dynamic functional states

The first widely used PDAC CAF framework separated α-SMA-high myofibroblastic CAFs (myCAFs), located close to malignant glands, from cytokine-secreting inflammatory CAFs (iCAFs), which were more distant in experimental models (13). Single-cell analysis later identified antigen-presenting CAFs (apCAFs) expressing major histocompatibility complex class II (MHC II) genes and CD74 (14). These categories created a common language for CAF studies and linked phenotype to spatial position and signaling.

Further work has complicated this three-part scheme. LRRC15-positive myofibroblasts, senescent CAFs, interferon-response CAFs and complement-secreting CAFs have been linked to different immune and clinical outcomes (15–19). Pan-cancer and multiomic studies have also proposed broader groups based on steady-state, mechanical and immunomodulatory programs (20, 21). The growing list does not necessarily mean that each study has found a new stable cell type. Differences in platform, sampling, tumor stage, treatment and clustering resolution can divide a continuous biological spectrum in different ways.

Mechanistic perturbations in PDAC model systems support this interpretation. IL-1-driven JAK/STAT signaling promotes an inflammatory program, whereas transforming growth factor β (TGF-β) suppresses IL-1 receptor expression and favors myofibroblastic differentiation (22). Hypoxia can strengthen an iCAF-like program, and inhibition of macropinocytosis can drive a myCAF-to-iCAF shift through metabolic stress and MEK-ERK signaling (23, 24). These experimentally induced changes show that at least part of CAF heterogeneity reflects state rather than fixed fate.

The terms subtype and state remain useful if their meanings are kept distinct. Subtype describes a reproducible cluster or marker-defined group in a given dataset. State describes a functional phenotype that can be entered, maintained or left in response to local conditions. Some CAF differences may still reflect stable developmental lineages. The practical task is not to replace every subtype name, but to determine which features are stable, which are reversible and which predict an intervention response.

### 1.4 Scope and organizing principle of this review

This narrative review synthesizes mechanistic, single-cell, spatial and clinical studies of CAFs in PDAC, with targeted updates through September 2026. It does not attempt a systematic meta-analysis. Primary studies support mechanistic and clinical claims, whereas reviews provide conceptual context. Evidence is identified by species, experimental design and clinical maturity rather than pooled as if all studies tested the same biological question (9, 25).

Our central argument is that CAF heterogeneity in PDAC is best understood as a set of plastic, context-dependent functional states. Canonical subtype labels remain useful entry points, but they become clinically informative only when combined with location, treatment history, tumor-cell state and measurable function. This view also explains why broad stromal depletion has failed: a marker may cover both harmful and protective fibroblasts, and removal of one compartment can cause another to expand. The following sections develop this argument from origin and classification to state regulation, immune effects, clinical use and treatment design.

## 2 Origin and classification of PDAC CAFs

### 2.1 Cellular origins of CAFs

Resident pancreatic mesenchymal cells are the main source of CAFs in current mouse lineage-tracing models. Pancreatic stellate cells and other fibroblast populations are present around acini, ducts and vessels in the normal organ, where they support matrix homeostasis and tissue repair. In cancer, oncogenic epithelial signals and inflammatory injury activate these cells, expand selected populations and alter their differentiation. Lineage tracing has shown that Gli1-positive fibroblasts, although rare in normal pancreas, expand during carcinogenesis (26). A broader developmental analysis traced most resident pancreatic fibroblasts and tumor CAFs to splanchnic mesenchyme, with limited contribution from bone marrow or epithelial cells in the tested mouse model (27).

The lineage evidence argues against assigning every CAF state to a different extra-pancreatic source. A common developmental pool can generate substantial diversity under local instruction. This conclusion remains model-dependent. Genetic tracing labels cells according to promoter activity and developmental timing, and human tumors cannot be tested with the same methods. Bone marrow-derived fibrocytes, perivascular cells and mesothelial cells may contribute in selected settings even if they are not the dominant source.

Mesothelial origin is particularly relevant to apCAFs. Mouse lineage and functional studies indicate that mesothelial cells can enter tumors, downregulate mesothelial features and acquire an apCAF program. These cells can present antigen to CD4-positive T cells and promote regulatory T-cell expansion in experimental PDAC (28). More recent spatial work suggests that apCAF labels may cover at least two populations with different origins and neighborhoods (29). Origin can bias a cell toward a state without fully determining its function.

CAF heterogeneity can also be organized into lineages within the pancreas. CD105 expression separates fibroblast populations with distinct tumor effects in mouse models. CD105-negative fibroblasts supported adaptive antitumor immunity and restricted tumor growth, whereas CD105-positive cells were more permissive (30). Other work has shown nonredundant contributions from mesenchymal lineages to the PDAC stroma (31). The combined evidence suggests a layered model: developmental origin sets a range of possible responses, while tumor signals, location and stress determine the active state.

### 2.2 Canonical CAF states

#### 2.2.1 Myofibroblastic CAFs

myCAFs express contractile and ECM-associated genes, including ACTA2, TAGLN and selected collagens. They are often located next to malignant glands, consistent with TGF-β and Hedgehog signaling in experimental systems (13, 22, 32). Their functions include matrix deposition, collagen organization, tissue contraction and control of interstitial mechanics. These activities can increase stiffness and activate integrin-dependent signaling, but they cannot be assumed to promote cancer in every context.

Several observations show why myCAF abundance is an incomplete functional readout. Deleting type I collagen from α-SMA-positive myofibroblasts accelerated PDAC and increased immune suppression in mice (11). Depletion of regulatory T cells (Tregs) also reduced a myCAF compartment and unexpectedly accelerated carcinogenesis through compensatory myeloid recruitment (33). Conversely, LRRC15-positive and senescent myofibroblasts have been linked to immune suppression (15, 16). The myCAF label includes both protective and harmful programs. Matrix composition, cell age, signaling history and spatial relation to immune cells provide needed resolution.

#### 2.2.2 Inflammatory CAFs

iCAFs express cytokines and chemokines such as IL6, LIF, CXCL1 and related inflammatory programs. They were first distinguished from myCAFs by their lower α-SMA expression, secretory phenotype and greater distance from cancer cells (13). In experimental PDAC fibroblasts, IL-1 induces LIF and JAK/STAT signaling to support this state, whereas TGF-β antagonizes it (22). Other inputs, including hypoxia, oncostatin M from macrophages, pancreatic polypeptide and fibroblast-intrinsic NF-κB activation, can converge on inflammatory CAF programs (24, 34–36).

iCAFs can support tumor growth, myeloid recruitment and treatment resistance. IL6-mediated differences between CAF populations have been linked to therapy response (37), and inflammatory signaling can promote epithelial plasticity. Yet an inflammatory transcriptome alone does not define a uniform effect. Complement-secreting CAFs overlap transcriptionally with iCAFs but were associated with more favorable features in a recent multiomic study (19). Interpretation of an iCAF score depends on the genes driving it and on the surrounding immune and tumor-cell states.

#### 2.2.3 Antigen-presenting CAFs

apCAFs were identified by expression of MHC II genes and CD74 without the full costimulatory program of professional antigen-presenting cells (14). In murine functional assays, they can process and present antigen to CD4-positive T cells. The first interpretation emphasized immune regulation and possible induction of nonproductive or suppressive T-cell responses. Mesothelial-derived apCAFs were later shown to expand regulatory T cells in PDAC models (28).

This function is not universal across tumors or niches. Cross-cancer spatial analysis has separated mesothelial-related apCAFs near cancer cells from fibrocyte-related apCAFs in lymphocyte-rich regions, with both groups sharing SPP1 expression but occupying different cellular neighborhoods (29). In a non-PDAC cancer context, apCAFs near tertiary lymphoid structures enhanced T-cell activity and predicted immunotherapy response (38). These data caution against assigning an immune effect from MHC II expression alone. Origin, costimulatory capacity, antigen context and neighboring immune cells must be measured before apCAFs can be used as a therapeutic or predictive category.

### 2.3 Recently described CAF states

#### 2.3.1 LRRC15-positive CAFs

LRRC15 marks a TGF-β-associated myofibroblastic program found across solid tumors. Mouse perturbation experiments showed that LRRC15-positive fibroblasts establish a stromal setting that limits antitumor immunity, whereas clinical associations with checkpoint response require attention to tumor type (15). In PDAC, this state is useful because it connects a surface-associated marker to an immune phenotype. It is not exclusive to all suppressive CAFs, and it overlaps with broader myCAF programs. Its value will depend on whether protein-level detection can reproducibly identify the relevant cells in clinical tissue.

#### 2.3.2 Senescent CAFs

Senescence defines a functionally important subset of myofibroblastic CAFs. Senescent CAFs accumulate near tumor ducts during PDAC progression and express immune-regulatory factors. Genetic or pharmacologic removal of senescent stromal cells reduced suppressive macrophage activity, delayed tumor growth and improved treatment response in mouse models (16). A separate study found that senescent CAFs restricted CD8-positive T-cell activation and reduced immunotherapy responsiveness (17).

Senescence is a cellular program rather than a lineage. Its markers vary, and no single marker is sufficient. Senolytic treatment may also affect senescent epithelial, endothelial or immune cells. Clinical translation requires a composite definition of cell identity, stable cell-cycle arrest and secretory phenotype. The targeted senescent population must also be shown to be harmful in the treated setting.

#### 2.3.3 Interferon-response CAFs

A recent analysis of FAP-positive mesenchymal cells identified interferon-response programs in mouse and human PDAC, with tumor-restraining effects demonstrated experimentally (18). Because this protective population falls within a compartment often targeted for depletion, FAP expression cannot be equated with a single function. The state requires validation in independent PDAC cohorts, and its stability under therapy is not yet known.

#### 2.3.4 Complement-secreting CAFs

Complement-secreting CAFs express several complement pathway genes and show transcriptional and epigenetic overlap with iCAFs. Their proportion declined in the studied progression datasets, and higher representation was associated with better prognosis in the reported human analyses (19). Trajectory inference suggested a possible transition toward iCAFs, and experimental reprogramming was explored. Because trajectory analysis infers rather than directly observes cell movement, lineage and intervention studies are needed before this transition can be treated as established.

### 2.4 Heterogeneity and plasticity: subtype or state?

The subtype framework remains useful for communication, experimental isolation and comparison. Its weakness appears when cluster identity is mistaken for a stable biological unit. Single-cell clusters depend on tissue dissociation, sequencing depth, sample composition and analysis choices. Spatial proteomics and transcriptomics add location but use different marker panels and resolutions. Cross-study classification systems can identify shared mechanical, inflammatory and steady-state programs without removing tissue-specific variation (20, 21).

Evidence for lineage, state change and reversibility should be distinguished. Genetic lineage tracing establishes ancestry in the labeled mouse population, but shared ancestry alone does not demonstrate interconversion. Perturbation followed by repeated phenotyping supports an induced change in the experimental system; withdrawal or rescue is needed to establish reversibility. Pseudotime, RNA velocity, transcriptional similarity and spatial coexistence generate hypotheses about transitions but do not directly establish that an individual fibroblast changed state. Serial human samples can reveal treatment-associated remodeling, although altered abundance may reflect selection, recruitment or differential survival rather than conversion (2, 4, 22, 23, 27).

A constrained state space offers a more practical model than either a flat continuum or a rigid tree. Developmental origin and chromatin state limit the available responses, while local signals, matrix mechanics, metabolism and therapy select among them. Marker panels may therefore perform differently across disease stages and treatments. Functional axes such as matrix production, inflammatory signaling, antigen presentation and stress response may prove more reproducible. This model changes the therapeutic question from “Which subtype should be removed?” to “Which function is harmful in this patient, and which cells sustain it?” **Table 1** compares the major CAF populations within this framework.

**Table 1:** CAF programs in PDAC with spatial, plasticity and human-evidence annotations.

CAF program and markers	Principal output and context	Spatial niche	Plasticity or reversibility	Human evidence and limitations	References
myCAF α-SMA/ACTA2, TAGLN, ECM genes	Contractility and matrix production; TGF-β/Hedgehog-responsive. Mouse collagen perturbations show context-dependent restraint.	Often gland-adjacent; distribution varies with treatment and assay.	Cytokine-dependent switching in culture and metabolic perturbation in models; universal *in vivo* reversibility unproven.	Repeated human detection and spatial correspondence; α-SMA alone has no uniform functional meaning.	(11, 13, 14, 22, 23, 32, 33)
iCAF IL6, LIF, CXCL1, JAK/STAT	Inflammatory secretion under IL-1, hypoxia, OSM and related inputs; effects depend on the circuit.	Often distal to glands; inflammatory programs can coexist with matrix programs.	Inducible in cytokine and nutrient-stress models; patient-level conversion is not established by pseudotime.	Human expression and spatial evidence; some distal inflammatory/complement features associate with favorable outcome.	(13, 14, 22–24, 34–37, 80)
apCAF MHC II, CD74; variable SPP1	Antigen presentation; murine mesothelial-derived cells can expand Tregs.	Tumor-adjacent or lymphocyte-rich niches; lineage/context dependent.	Phenotypic change supported in mouse models; all apCAF lineages should not be assumed interconvertible.	Human detection supports relevance; MHC II does not establish origin, costimulation or immune benefit.	(14, 28, 29, 38)
LRRC15-positive CAF TGF-β-associated myCAF program	Suppressive stromal function established by mouse perturbation.	Activated myofibroblastic stroma; overlap with myCAFs.	TGF-β-responsive; durability and reversal in treated human PDAC unresolved.	Human/pan-cancer associations; no validated PDAC treatment-selection assay.	(15)
Senescent CAF Composite arrest and secretory markers	Persistent stress program; senolysis improves immunity in mouse PDAC.	Duct-proximal enrichment in reported PDAC studies.	Senescence is potentially persistent; senolysis is cell removal, not proof of reversal.	Human tissue observations plus mouse causality; no single specific marker or established clinical senolytic selection rule.	(16, 17)
Interferon-response CAF IFN-response genes within FAP-positive cells	Tumor-restraining experimental activity.	Detected within PDAC stroma; universal spatial criterion not established.	Regulation can be tested experimentally; longitudinal human stability unknown.	Mouse and human profiling; independent clinical utility and intervention benefit unresolved.	(18)
Complement-secreting CAF Complement gene programs	Overlap with iCAFs; favorable human associations.	Progression-associated distribution; not defined by one universal niche.	Trajectory suggests a relationship to iCAFs; perturbation does not prove spontaneous human conversion.	Human multiomic and retrospective prognosis evidence; prospective validation absent.	(19, 80)
Meflin-positive CAF Meflin/ISLR	Tumor restraint and regulation of stiffness in mouse studies.	Stellate/fibroblast compartment; niche and abundance vary with progression.	Retinoid induction demonstrated in models; long-term human persistence uncertain.	Favorable human association with causal mouse support; no established selection assay.	(70, 92)

We propose an operational distinction between a molecular state, a clinically relevant state and a clinically actionable state. A molecular state is a reproducible expression or functional program. Clinical relevance additionally requires reproducibility across independent patient cohorts and platforms, localization to a defined tissue niche, and an association with a clinically meaningful endpoint after accounting for major confounders. A state can be prognostically relevant without being a causal therapeutic target. Candidate actionability requires functional perturbation linking the state to a treatment-limiting mechanism, a measurable vulnerability accessible to an intervention, and characterization of persistence or reversibility under the intended treatment. Validated actionability further requires prospective evidence that using the state assay improves treatment selection or patient outcome. These are proposed evaluation criteria, not a validated scoring system.

Each state should therefore be reported with an identity panel, functional output, spatial niche, species and model, treatment history, transition evidence and clinical evidence category. Reversibility is an experimentally testable attribute rather than an assumption or a prerequisite for every target; a persistent harmful state may instead justify selective depletion. **Table 1** makes these distinctions explicit.

### 2.5 Cross-species correspondence and its limits

Mouse and human observations answer complementary questions. Genetically engineered and transplantation models permit lineage tracing and causal perturbation, whereas human single-cell and spatial studies establish whether comparable programs occur in patient tissue. Shared myofibroblastic, inflammatory and MHC II-associated programs support cross-species correspondence (13, 14). Pan-cancer multiomics further identifies conserved mechanical and immunomodulatory programs (21). This supports conservation at the program level, but does not establish identical cell ancestry, state frequencies, spatial organization or therapeutic effects in human PDAC.

Cross-species mapping should compare orthologous gene modules within independently identified fibroblasts, confirm protein expression and spatial position, and retain species-specific or unmatched populations. Matching a cluster name or integrating datasets computationally cannot establish evolutionary homology by itself. Disease stage, anatomical site, treatment exposure and model type should be matched where possible. Mouse tumors arise over different timescales and in different immune and genetic backgrounds; xenograft stroma may become host-derived, while culture can alter fibroblast identity. **Table 2** separates mouse perturbation from human observational evidence and specifies what remains uncertain. Putatively shared states should consequently be described as conserved programs with model-dependent functions rather than interchangeable therapeutic targets (14, 21, 39).

**Table 2:** Cross-species comparison of CAF programs and evidence boundaries.

Program or question	Mouse/model evidence	Human evidence	What is shared and what is unresolved	References
Canonical myCAF/iCAF programs	Co-culture and perturbation establish cytokine-dependent phenotypes.	Single-cell and spatial detection of related contractile and inflammatory programs.	Program-level correspondence is supported; identical frequencies, spatial distances and outcomes are not implied.	(13, 14, 22, 80)
apCAF origin and function	Mesothelial lineage tracing and antigen-specific T-cell experiments support an origin and regulatory function.	MHC II/CD74-positive fibroblasts and distinct spatial niches are observed.	Shared presentation program; human lineage ancestry and immune direction cannot be inferred from MHC II alone.	(14, 28, 29)
Mechanical and immune programs across cancers	Multiomic and perturbation studies identify mechanoresponsive and immunomodulatory programs.	Related programs occur across human tumors; spatial studies resolve recurring neighborhoods.	Conservation of modules supports comparison; pan-cancer similarity is not PDAC-specific therapeutic validation.	(20, 21, 39)
Tumor restraint	Collagen deletion, Meflin perturbation and CD105-defined fibroblast experiments can establish causality.	Meflin and other stromal features associate with patient outcome.	Mouse causal restraint and human association are complementary; clinical benefit from preserving each population remains unproven.	(11, 30, 70)
Senescence and interferon response	Mouse perturbations support suppressive senescent programs and tumor-restraining interferon-response activity.	Related CAF programs are detected in human PDAC.	Human detection supports relevance; therapeutic selectivity, reversibility and net clinical effect require testing.	(16–18)
Complement-associated states	Perturbation models can test reprogramming and function.	Multiomics and retrospective prognosis associate complement programs with favorable features.	An inferred trajectory does not establish spontaneous conversion in either species.	(19, 80)
Treatment-associated remodeling	Controlled exposures enable temporal perturbation and mechanism tests.	Neoadjuvant cohorts show altered stromal composition and interactions; paricalcitol trials provide serial-biopsy evidence.	Both show an exposure-associated ecosystem response. Induction versus selection and efficacy require separate evidence.	(4, 5, 23, 58, 84, 93)

The comparison separates causal perturbation from human detection and association. Model evidence includes genetically engineered mice, transplantation and culture systems as specified in the cited studies. Conserved programs do not establish evolutionary identity, equal abundance or equivalent clinical response.

## 3 Regulatory mechanisms shaping CAF states

### 3.1 Tumor-derived and heterocellular signals

#### 3.1.1 TGF-β signaling

TGF-β is a major driver of myofibroblastic differentiation. In PDAC fibroblast models, it induces contractile and ECM programs and suppresses IL-1 receptor expression, thereby limiting the inflammatory state (22). Matrix storage, integrin-mediated activation and feedback from immune cells also regulate TGF-β activity. High pathway activity may reflect both ligand production and tissue mechanics.

Its effects depend on context. TGF-β can support an LRRC15-positive suppressive program, but it can also maintain myofibroblasts with tumor-restraining functions (15, 33). Systemic pathway blockade affects cancer and immune cells as well as CAFs. A CAF-directed TGF-β strategy must specify the downstream function being targeted and monitor compensatory inflammation or myeloid recruitment.

#### 3.1.2 IL-1/JAK/STAT and related inflammatory circuits

In experimental PDAC, cancer-derived IL-1 activates LIF and JAK/STAT signaling in fibroblasts, generating an iCAF program that is opposed by TGF-β (22). This axis provides a mechanistic basis for the spatial separation of myCAFs and iCAFs because ligand concentration and receptor expression vary with distance from malignant glands. It also explains why pathway inhibition can change composition rather than simply reduce CAF activity.

Several heterocellular signals reinforce the same inflammatory output. Model perturbations show that macrophage-derived oncostatin M acts through its receptor OSMR on fibroblasts to promote inflammatory gene expression, tumor growth and metastasis (34). Tumor and neutrophil signaling can form a CXCL1-TNF circuit that sustains inflammatory CAF polarization and T-cell exclusion (40). Feedback among three or more cell types can maintain an inflammatory CAF state, so blocking one cytokine may be insufficient unless the sustaining loop is identified.

#### 3.1.3 Hedgehog signaling

Hedgehog ligands are produced mainly by cancer cells and act on stromal cells. In PDAC models, pathway activity is enriched in myCAFs, Sonic Hedgehog promotes a myofibroblastic composition, and inhibition shifts the balance toward inflammatory CAFs (32). These effects help reconcile earlier contradictory studies. Reduced matrix can improve drug access in one setting while loss of a restraining myofibroblastic compartment can worsen biology in another.

The clinical result is cautionary. Adding the Hedgehog inhibitor vismodegib to gemcitabine and nab-paclitaxel did not improve efficacy in a phase 2 metastatic PDAC study (41). This negative result leaves Hedgehog biology unresolved but shows that pathway expression and stromal abundance did not guide treatment adequately. Future approaches would need a state-resolved pharmacodynamic marker and a combination designed around the induced CAF shift.

#### 3.1.4 Other regulatory inputs

CAF states also respond to MAPK activity, IL-33, pancreatic polypeptide, neural signals and noncoding RNA programs. Epithelial MAPK signaling can promote myofibroblastic differentiation and link basal-like cancer niches to myCAF features (42). Oncogenic KRAS-dependent IL-33 production reshapes stromal and immune interactions, although IL-33 can support different immune outcomes according to the responding cells and tissue setting (43–45). Schwann cells can push fibroblasts toward inflammatory states while also affecting malignant-cell identity (46). These examples show that CAF regulation is distributed across the TME rather than controlled by one master ligand.

### 3.2 ECM composition and mechanics

The ECM regulates CAF state through stiffness, ligand presentation and force transmission. Integrins, focal adhesion kinase (FAK), Src-family kinases and mechanosensitive transcriptional programs connect matrix organization to cell behavior. CAFs both create and sense this environment, forming a feedback loop in which matrix deposition raises tension and tension reinforces a contractile state. Multiomic analyses across tumors have identified conserved mechanoresponsive CAF programs, supporting this regulatory axis (21).

Composition matters as much as bulk density. Type I collagen produced by α-SMA-positive myofibroblasts can restrain PDAC, whereas a cancer-cell-derived collagen I homotrimer promotes oncogenic signaling, immune suppression and an altered microbiome (11, 47). Intact and matrix metalloprotease-cleaved collagen can also have opposing effects through DDR1-dependent signaling and tumor metabolism (48). Cancer-cell-derived fibrillar collagen processing has shown additional suppressive effects (49). These studies indicate that “collagen-high” is not a sufficient biological category. Source, molecular form, organization and proteolysis must be specified.

This distinction has direct therapeutic implications. Enzymatic matrix removal may decompress vessels, but it can also remove physical restraints or create bioactive fragments. The negative phase 3 result for pegvorhyaluronidase alfa in hyaluronan-high metastatic PDAC confirms that selecting patients by matrix abundance alone does not ensure clinical benefit (50). Matrix-directed therapy should be linked to a functional consequence, such as perfusion, immune exclusion or a defined receptor pathway, and should include early evidence that the expected tissue change occurred.

### 3.3 Metabolic stress and stress-adapted CAF states

Stress adaptation provides a useful organizing framework for CAF plasticity. Baseline heterogeneity describes the distribution measured before a specified experimental or therapeutic exposure; untreated tumors are themselves exposed to hypoxia, nutrient scarcity and inflammatory stress. Stress-induced states arise when these pressures alter fibroblast function, whereas treatment-selected states become enriched because pre-existing cells persist or expand. Both processes can coexist. The stress-adaptation synthesis of Hu et al. links these pressures to immune suppression and resistance across solid tumors; it is used here as a conceptual review, with PDAC-specific mechanisms supported by primary studies (8, 16, 23, 24).

PDAC contains regions with low oxygen and limited nutrients. CAFs adapt through autophagy, macropinocytosis and metabolic exchange, and these adaptations can stabilize particular states. Pancreatic stellate cells release alanine through autophagy, providing carbon for tumor metabolism (51). In PDAC model systems, CAF macropinocytosis supplies amino acids to fibroblasts and neighboring cancer cells during glutamine deprivation (52). More recent work suggests that this pathway also maintains myCAF identity, because its inhibition produces metabolic stress and a shift toward an inflammatory state (23).

Additional exchanges involve glutamine and glutamate, asparagine, lactate and lipids. NetG1-positive CAFs support tumor survival through glutamate and glutamine metabolism and suppress natural killer cell killing (53). CAF-derived lysophosphatidylcholines help cancer cells maintain membrane lipid homeostasis during hypoxia and nutrient scarcity (54). Cancer-derived lactate can be reused by CAFs to maintain fibrotic and immunosuppressive functions (55). Metabolism supplies fuel while also regulating CAF identity and immune function.

In PDAC experimental systems, hypoxia potentiates cytokine-driven inflammatory fibroblast programs (24), whereas nutrient scavenging through macropinocytosis helps maintain myCAF identity during metabolic stress (23). These observations connect oxygen and nutrient supply to the balance of matrix-producing and secretory functions. Senescence adds a distinct, potentially persistent stress response with an altered secretome and impaired immune surveillance in mouse PDAC (16, 17). Stress adaptation is therefore an axis that can intersect canonical CAF programs, not evidence for a single additional subtype or universal tumor-promoting fate.

Stress-directed interventions should measure both fibroblast survival and state change. A decrease in a myCAF score after macropinocytosis inhibition does not by itself prove depletion, and enrichment of inflammatory markers need not imply a worse therapeutic outcome (23). Washout, rescue and repeated functional assays can distinguish transient adaptation from a persistent state. In patients, these tests must be complemented by tissue measurements because metabolic dependencies vary across niches and systemic inhibitors also affect malignant and immune cells.

### 3.4 Therapy-induced remodeling

Therapy changes the signals that maintain CAF states. Human spatial transcriptomics identified altered CAF-malignant cell interactions after neoadjuvant treatment, including enrichment of IL6-family signaling that conferred chemotherapy resistance in functional models (4). Single-cell analysis of treated human PDAC also found major changes in malignant and stromal composition, with features that may further limit immunotherapy (5). These data show that the post-treatment stroma is not equivalent to untreated tissue.

Treatment can create new selection pressures. Chemotherapy induces placental growth factor and VEGF-family signaling that activates collagen-producing CAFs in preclinical models (56). Combined MEK and STAT3 inhibition enriches CAFs with mesenchymal stem cell-like features and changes immune exclusion, illustrating how pathway blockade can uncover latent stromal plasticity (57). In mouse PDAC models, KRAS inhibition also remodels fibroblast and immune compartments while selecting cancer-cell states that support relapse (58–60).

Treatment-induced remodeling should not automatically be called cellular transition. Human comparisons before and after neoadjuvant therapy establish an exposure-associated change in the sampled ecosystem (4, 5). Distinguishing induction from selection requires paired sampling, absolute cell-density measurements and proliferation or death readouts, supported where feasible by lineage-resolved perturbation in models. A treatment-enriched CAF cluster may represent survival of a baseline minority rather than a newly generated state. This distinction determines whether an intervention should prevent adaptation, eliminate a persistent population or interrupt its downstream circuit.

A baseline state assay should therefore be linked to an on-treatment readout and a predefined sampling time. Serial biopsy where feasible, spatial imaging and functional model studies can determine whether the target persists and whether the intended mechanism changes. **Figure 1** summarizes experimentally supported state changes separately from inferred or context-dependent relationships.

**Figure 1:**
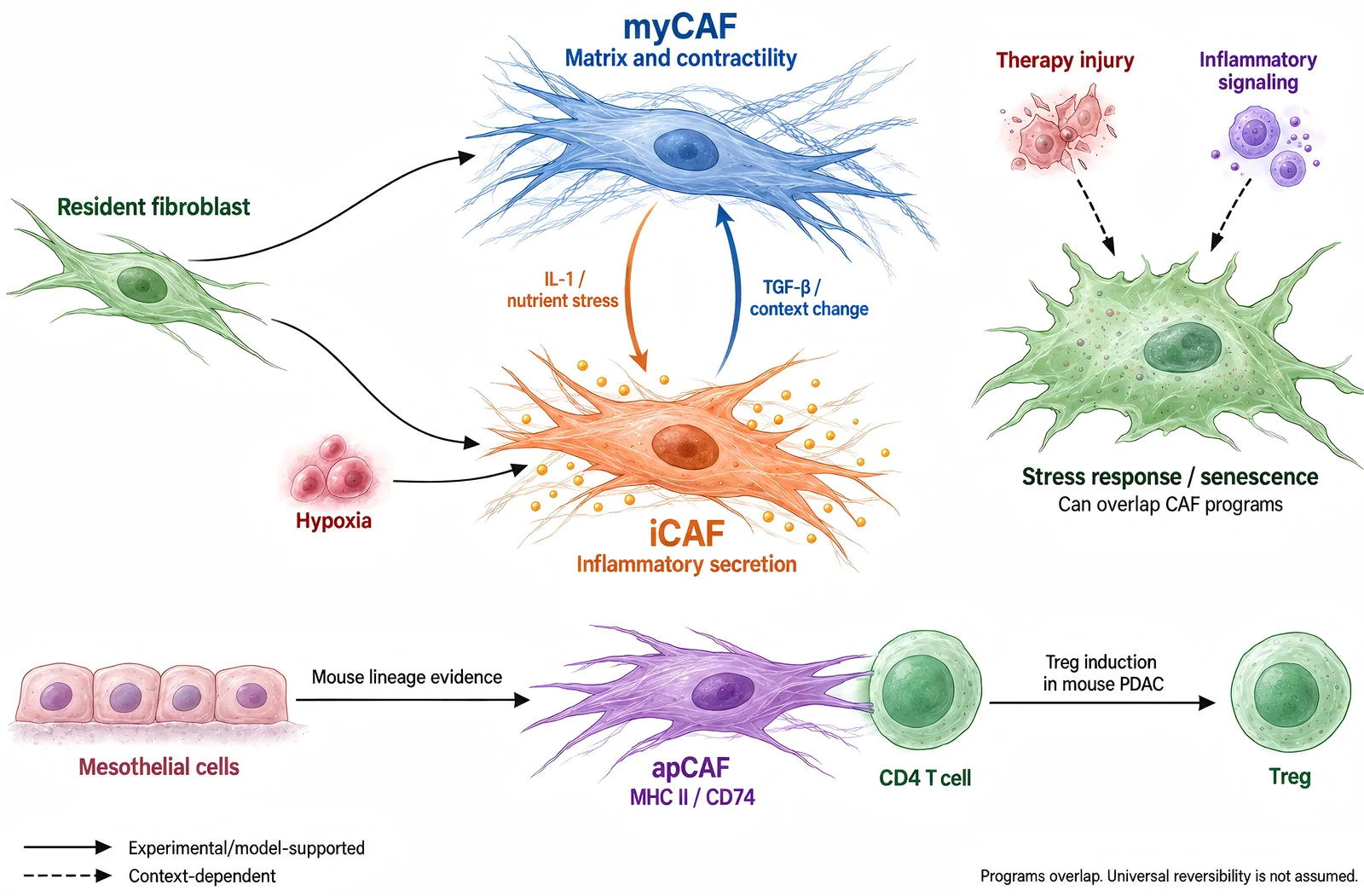
Context-dependent CAF programs and evidence for state change. Resident fibroblast and mesothelial contributions are shown separately from functional programs. The myCAF-iCAF relationship is supported by cytokine and metabolic perturbations in experimental PDAC; opposing directional arrows indicate context-dependent changes rather than universal reversibility in patients. Hypoxia and nutrient stress alter inflammatory and matrix-associated output. Therapy can induce adaptation or select pre-existing cells. Dashed relationships indicate proposed or model-dependent changes and do not imply lineage-traced conversion. Senescence is a potentially persistent stress response, and no reversal is assumed. Antigen-presenting CAF function depends on origin and immune niche. The diagram synthesizes the cited evidence and is not a measured lineage tree (13, 14, 16, 17, 22–24, 28). CAF, cancer-associated fibroblast; myCAF, myofibroblastic CAF; iCAF, inflammatory CAF; apCAF, antigen-presenting CAF; ECM, extracellular matrix; TGF-β, transforming growth factor β; IL-1, interleukin-1; MHC II, major histocompatibility complex class II.

## 4 Functional roles of CAFs in PDAC progression

### 4.1 ECM organization and tumor growth

CAFs are the main producers and organizers of the PDAC ECM, but their effects depend on more than the amount of fibrosis. Matrix architecture controls tissue tension, diffusion, vascular compression and the availability of bound growth factors. Crosslinked collagen and fibronectin can activate integrin, FAK and YAP/TAZ signaling in cancer cells, whereas aligned fibers can provide tracks for invasion. CAF metabolism also feeds matrix production. Proline synthesis through PYCR1 supports collagen deposition in activated fibroblasts (61). Most direct functional evidence comes from breast cancer models, however, and requires validation in PDAC.

PDAC-specific collagen studies reveal a complex balance. In mouse PDAC, loss of collagen I from α-SMA-positive myofibroblasts accelerated tumor progression and increased suppressive immune features (11). Similar experiments in liver metastasis models showed that fibroblast-derived type I collagen could oppose other tumor-promoting CAF effects (62). By contrast, cancer-cell-derived collagen I homotrimers supported tumor growth, altered immunity and reduced response to PD-1 blockade (47). Collagenolysis created DDR1-dependent signals that affected nutrient use and outcome (48). Matrix source and processing can reverse biological effect even when the protein name is unchanged.

ECM proteins also act as temporal signals. Proteomic profiling identified nidogen-2 as a matrix component that changes during progression and modulates fibrosis and therapy response in preclinical PDAC (63). A useful matrix biomarker may therefore need to measure an active process, such as crosslinking, cleavage or receptor engagement, rather than total stain area. This distinction may explain why bulk stromal depletion has not produced consistent benefit.

### 4.2 Invasion and metastasis

CAFs promote invasion through physical remodeling and paracrine signaling. Contractile fibroblasts reorganize ECM, open migration paths and increase local stiffness. Inflammatory CAFs secrete factors that activate survival and motility pathways in cancer cells. Macrophage-derived oncostatin M can reprogram CAFs, and the resulting fibroblasts promote tumor growth and metastasis in mouse models (34). Neural-stromal interactions may add another layer, because Schwann cells alter both fibroblast and cancer-cell states (46).

The metastatic setting is not simply a copy of the primary tumor. Single-cell analysis of primary and metastatic human PDAC found metabolic reprogramming in metastasis-associated fibroblasts, including high UPP1 expression, together with deeper T-cell exhaustion (64). Spatial mapping of metastatic disease revealed extensive regional plasticity and different cellular neighborhoods (65). CAFs at metastatic sites are shaped by the resident mesenchyme and immune environment of the target organ, so primary-tumor markers may not predict their function.

CAF effects on invasion can also be indirect. Integrin and growth-factor signaling promotes epithelial-mesenchymal transition (EMT), while matrix and cytokine gradients select motile cancer-cell states. Human single-cell data integrated with organoid-CAF co-culture linked CAF density to inflammatory and EMT programs in epithelial cells. The analysis also identified ITGB1 and VEGF-A/NRP1 interactions as candidate mediators (66). The co-culture experiments support causality more strongly than transcriptomic association alone, although they simplify the full immune and vascular context.

### 4.3 Metabolic support and survival under stress

The poorly perfused PDAC stroma creates metabolic scarcity, yet CAFs help malignant cells survive it. Autophagic alanine secretion by pancreatic stellate cells provides carbon that supports tumor-cell mitochondrial metabolism (51). Macropinocytosis allows CAFs to scavenge extracellular protein, maintain their own fitness and release amino acids for cancer cells (52). NetG1-positive CAFs coordinate nutrient support with immune suppression through glutamatergic signaling (53). CAF-derived lipids also help malignant cells maintain membrane composition under hypoxia (54).

These exchanges are reciprocal. Cancer cells release lactate that CAFs reuse to maintain fibrotic and suppressive programs (55). Nutrient stress alters CAF state, and the resulting state changes the metabolites supplied to cancer cells. This feedback connects tissue perfusion, metabolism and fibroblast identity. Blocking one metabolite may expose dependence on another pathway or change CAF composition.

Metabolic symbiosis also occurs among malignant subpopulations. Distinct PDAC cell states can exchange asparagine and resist therapy under respiratory stress (67). Although this mechanism is not CAF-specific, it matters for stromal targeting because CAF-derived nutrients can change the selective pressure among cancer-cell states. Combining a CAF metabolic target with a tumor-cell metabolic target may be necessary, but the combination should be based on measured flux or dependency rather than transcript expression alone.

### 4.4 Epithelial plasticity and subtype change

PDAC cancer cells occupy classical and basal-like states, with mixed and transitional states within individual tumors. CAFs can influence this balance. Transfer learning across human single-cell data and patient-derived co-cultures associated CAFs with EMT and inflammatory programs in epithelial cells (66). Patient-matched CAF and organoid models further showed CAF-driven EMT and a classical-to-basal shift, with effects that depended on the fibroblast and tumor pair (68). Together, these models link stromal state to malignant-cell identity.

The direction of change depends on context. Epithelial MAPK activity can promote myCAF differentiation, while CAFs feed back on epithelial signaling (42). KRAS inhibition rapidly changes malignant-cell composition and the TME. Classical cells can survive acute inhibition and seed relapse even though basal-like disease is usually linked to poor prognosis (58, 60). Static basal and classical labels do not fully predict treatment response. The more relevant variable may be the capacity to switch state under a specific pressure.

CAF-driven plasticity also intersects with DNA repair. A recent study combining human associations with mechanistic models found that CAF-secreted ECM activates integrin-Src-FAK and SGK-dependent NDRG1 phosphorylation, supporting R-loop processing and reducing DNA damage after chemotherapy (69). This mechanism connects matrix sensing to a nuclear resistance program and provides a specific example of how stromal contact can alter tumor-cell state beyond transcriptional subtype.

### 4.5 Tumor-restraining CAF states

Evidence for tumor-restraining CAFs is central to interpretation of the field. Meflin-positive fibroblasts were associated with favorable outcome in human PDAC, and loss or ablation of these cells produced less differentiated, more aggressive tumors in mouse models (70). In mouse experiments, CD105-negative pancreatic fibroblasts limited tumor growth through adaptive immunity (30). Interferon-response CAFs and complement-secreting CAFs have also been linked to restrictive functions or better outcome (18, 19).

Protective effects can arise from matrix production, immune support or maintenance of tissue organization. α-SMA-positive fibroblasts and their collagen can restrain aggressive growth (11, 33). Estrogen-dependent instruction of a tumor-suppressive stromal environment has recently been proposed, with sex-related differences in stromal biomarkers and fibroblast phenotype (71). These studies broaden the concept of restriction beyond one marker.

The evidence does not justify a universal protective CAF class. Some findings come from mouse genetics, others from human association, and several recent single-cell studies still lack broad external validation. A state that restrains early tumor growth might later support survival or repair after treatment. Current evidence supports a narrower conclusion: CAF functions are bidirectional and conditional. Therapeutic design should preserve or induce beneficial functions rather than assume that all activated fibroblasts should be removed.

## 5 CAFs and the immune tumor microenvironment

### 5.1 T cells and regulatory T cells

PDAC commonly excludes cytotoxic T cells from malignant glands and contains abundant regulatory T cells. CAFs contribute through chemokines, matrix organization, antigen presentation and metabolic competition. LRRC15-positive myofibroblasts establish a suppressive stromal program associated with weak antitumor immunity (15). Senescent CAFs limit CD8-positive T-cell activation and reduce response to immunotherapy in mouse models (16, 17). Dense or aligned matrix may also restrict movement, although physical exclusion is difficult to separate from chemokine and myeloid effects in human tissue.

apCAFs illustrate the context dependence of immune regulation. Their MHC II program permits antigen presentation, but lack of classical costimulation can favor ineffective or regulatory responses (14). Mesothelial-derived apCAFs expanded regulatory T cells in mouse PDAC (28). In lymphocyte-rich niches, however, antigen-presenting fibroblasts may support local immune activation. Spatial context and costimulatory state are therefore required to interpret an apCAF signal.

Regulatory T cells also regulate CAFs. Treg depletion in a PDAC mouse model unexpectedly removed TGF-β support for myCAFs, increased myeloid-recruiting chemokines and accelerated carcinogenesis (33). A cell usually considered suppressive can therefore maintain a protective stromal balance. Immune and stromal therapies cannot be designed independently.

### 5.2 Macrophages

CAFs shape macrophage recruitment and differentiation through cytokines, metabolites and cell-surface glycans. CAF sialylation, regulated in part by ST3GAL4, engages Siglec receptors and promotes differentiation of CD163-positive, CD206-positive macrophages *in vitro* (72). Stromal HIF2 signaling increases suppressive macrophage and Treg features; genetic or pharmacologic HIF2 inhibition improved survival and checkpoint response in PDAC models (73). Senescent CAFs also promote macrophage dysfunction (16).

The interaction is bidirectional. Macrophage-derived oncostatin M reprograms fibroblasts toward an inflammatory, tumor-promoting state (34). Resident pancreatic macrophages can drive fibrosis with different effects in inflammatory injury and PDAC (74). Origin and activation state matter on both sides of the interaction. Broad macrophage reduction may remove a fibrosis-inducing population while disrupting tissue-repair or antigen-presenting functions.

These loops create therapeutic opportunities. HIF2, OSM-OSMR and glycan-Siglec signaling provide more specific entry points than generic macrophage depletion. However, each pathway is used outside CAF-macrophage communication. Pharmacodynamic studies should confirm that the intended stromal circuit, rather than only a circulating cell count, has changed.

### 5.3 Neutrophils, myeloid-derived suppressor cells, dendritic cells and natural killer cells

Neutrophils and myeloid-derived suppressor cells are major barriers to T-cell entry in PDAC. A cancer-cell CXCL1 program recruits CXCR2-positive neutrophilic cells, and neutrophil-derived TNF reinforces CXCL1 production by cancer cells and CAFs, inflammatory CAF polarization and T-cell dysfunction (40). After neoadjuvant vaccine and PD-1 blockade, higher tumor-associated neutrophil density was associated with poorer survival, supporting clinical relevance for these circuits (6).

CAFs can also suppress natural killer cells. In functional model systems, NetG1-positive CAFs provided metabolic support to cancer cells and reduced NK-cell-mediated killing (53). Glycan-mediated interactions and ECM organization may further limit innate immune contact. Dendritic cells are often scarce or dysfunctional in PDAC, and apCAF antigen presentation may compete with professional antigen-presenting pathways. Direct CAF effects on dendritic cells remain less defined than effects on macrophages, neutrophils and T cells.

The IL-33 axis shows why immune-cell identity and timing must be specified. Tumor and stromal IL-33 can promote type 2 immunity and tumor growth, including through type 2 innate lymphoid cell (ILC2) responses (43, 44). In another setting, IL-33-activated ILC2s induced tertiary lymphoid structures and supported antitumor immunity (45). IL-33 is not intrinsically tumor-promoting or tumor-restraining. Its effect depends on tissue damage, the responding ILC2 state, lymphoid organization and other signals.

### 5.4 Immune exclusion and resistance to immunotherapy

Immune exclusion emerges from combined matrix, chemokine and myeloid programs rather than one barrier. Spatial studies show that CAF states and immune composition form recurring neighborhoods (2, 39). Treatment can shift these neighborhoods, and checkpoint blockade can induce compensatory neutrophil or regulatory programs (6). This helps explain why PD-1 or CTLA-4 inhibition alone has limited activity in unselected PDAC.

Preclinical combinations can convert the TME. Intratumoral TLR9 agonism promoted an immunopermissive transition and cooperated with anti-PD-1 therapy in mouse models (75). In PDAC mouse models, combined MEK and STAT3 inhibition altered CAF plasticity and reduced stromal inflammation, creating a more permissive T-cell environment (57). Inhibiting CAF autophagy changed desmoplasia and adaptive PD-L1 regulation, enabling immunochemotherapy in experimental models (76). These studies show that stromal intervention can support immunotherapy, but they also show that the mechanism and sequence of treatment matter.

A useful immune biomarker should integrate at least three features: CAF state, spatial T-cell access and the dominant suppressive myeloid circuit. CAF abundance alone cannot distinguish an LRRC15-rich suppressive niche from a Meflin-positive or interferon-response niche. Spatial multiplex imaging or focused transcript panels may provide this information with less tissue than whole-transcriptome profiling, but prospective validation is required. **Figure 2** summarizes the coupled matrix, metabolic and immune functions of CAFs.

**Figure 2:**
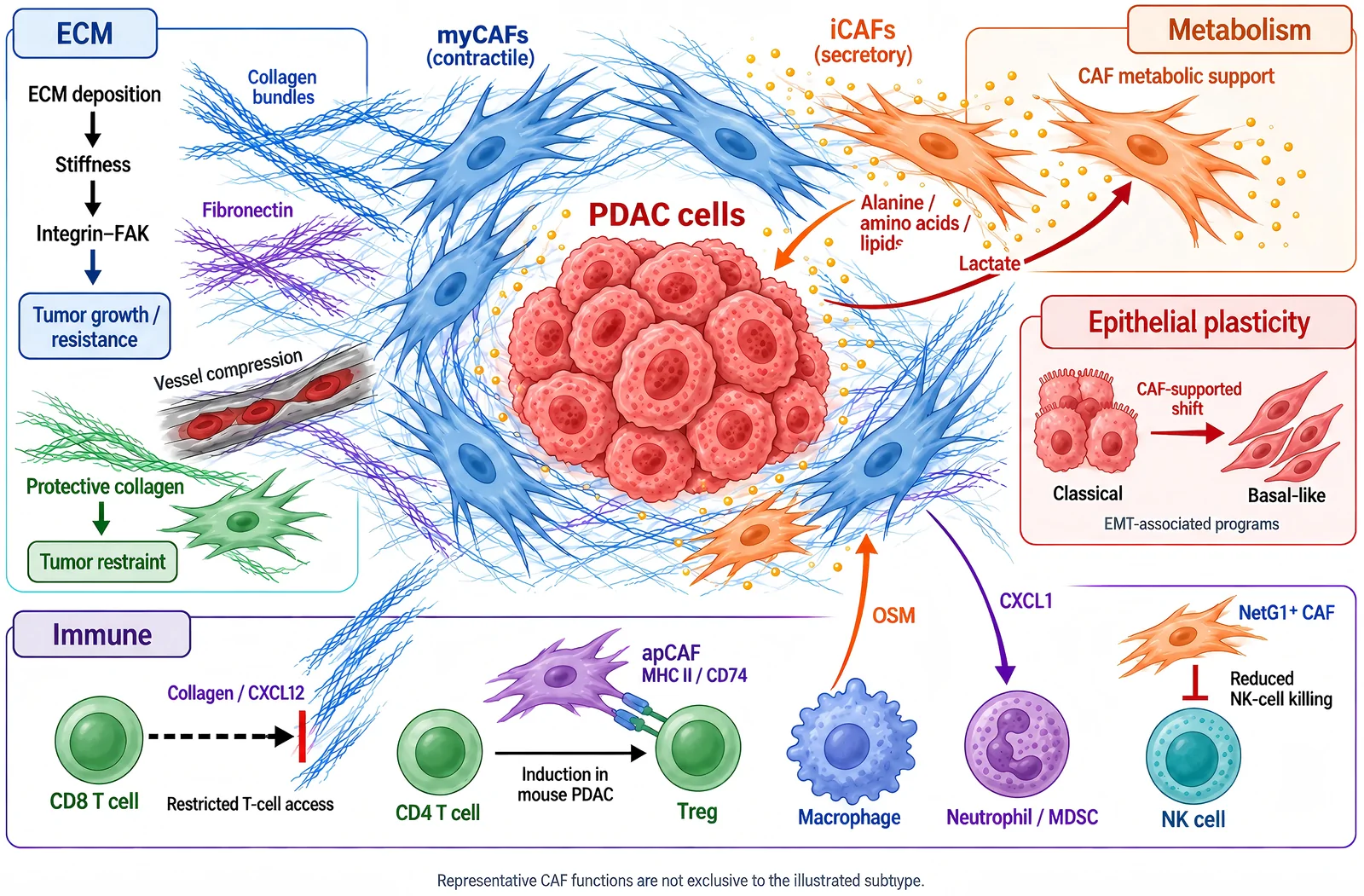
Coupled matrix, metabolic and immune functions of CAFs in PDAC. Contractile and secretory programs organize extracellular matrix and exchange signals or metabolites with malignant and immune cells. Matrix can influence vascular compression and integrin-FAK signaling, whereas selected collagen pools can restrain tumor growth. CAF-derived nutrients support cancer cells under stress, and reciprocal lactate use can sustain stromal functions. Cytokines, antigen presentation, matrix organization and NetG1-associated activity contribute to immune regulation. The schematic groups representative mechanisms and does not assign each output exclusively to the illustrated subtype. OSM also acts from macrophages toward CAFs; the grouped IL-6/OSM label denotes a reciprocal circuit, not proof that CAFs are the sole OSM source. Arrows summarize interactions, blunt ends indicate inhibition and the crossed path denotes restricted T-cell access. These mechanisms derive mainly from experimental systems, with human associations where discussed in Sections 4-5 (11, 14, 28, 34, 40, 51–55). PDAC, pancreatic ductal adenocarcinoma; ECM, extracellular matrix; FAK, focal adhesion kinase; EMT, epithelial-mesenchymal transition; OSM, oncostatin M; Treg, regulatory T cell; MDSC, myeloid-derived suppressor cell; NK, natural killer; NetG1, netrin G1; MHC II, major histocompatibility complex class II.

## 6 Clinical significance of CAF states

### 6.1 Prognostic biomarkers

CAF and matrix features are associated with outcome, but the direction varies with the feature measured. In tissue from the CONKO-001 trial, high α-SMA expression was linked to poorer disease-free and overall survival, while aspects of dense stromal quality showed a different association (77). Spatial analysis of matrix architecture and inflammatory cell interactions produced a prognostic signature that validated in an independent cohort (78). A classifier separating permissive and restrictive CAF patterns added prognostic information to tumor-intrinsic classical and basal-like subtypes (79).

Spatially resolved human data refine the prognostic interpretation. Croft et al. combined spatial profiling of resected PDAC with single-cell data and found different tumor-proximal and tumor-distal fibroblast programs. Higher podoplanin and hypoxia-associated expression tracked with poor outcome, whereas selected immune-regulatory and complement-associated features tracked with longer survival (80). The cohort was selected by survival extremes, so these associations require validation in unselected patients and do not show that altering either compartment improves survival. This also cautions against transferring a uniformly harmful interpretation of an inflammatory CAF label from a mouse model to every human spatial niche.

Several newer states have candidate clinical associations. Meflin-positive and complement-secreting CAFs were associated with better outcomes, whereas senescent, LRRC15-positive or selected inflammatory programs were linked to unfavorable biology (15, 16, 19, 70). Most results remain retrospective or supported mainly by models. These states require validation before they can become clinical tests.

Histology may offer a more scalable molecular surrogate. Bao et al. trained and externally validated a model that predicts a COL12A1/LOX/LOXL2 expression score from routine H&E slides, with independent spatial assessment (81). Although survival information informed gene selection, the principal validated endpoint was prediction of a stromal remodeling signature. The score is not a direct mechanical stiffness measurement, a CAF-exclusive readout or an established predictor of chemotherapy benefit. Prospective outcome validation remains necessary before it can guide treatment.

### 6.2 Predictive biomarkers

A prognostic biomarker identifies outcome independent of a specific treatment; a predictive biomarker identifies differential benefit from that treatment. This distinction is often blurred in CAF studies. A six-gene CAF-related risk score correlated with survival, immune infiltration and computational estimates of immunotherapy response, but such associations do not prove treatment prediction (82). Similarly, an immune-suppressive CAF signature may indicate poor outcome without showing that a CAF-directed drug improves it.

Treatment-linked human cohorts and patient-matched models identify candidate response mechanisms, but they do not by themselves validate a predictive biomarker. Spatial analyses linked CAF-malignant IL6-family signaling to treatment exposure and resistance in ex vivo systems (4). Matched organoid-CAF co-cultures demonstrated pair-dependent chemotherapy sensitivity (83). These findings support mechanistic prioritization; a clinical predictive claim requires a prespecified biomarker-by-treatment comparison in an appropriate comparative study.

Clinical trials illustrate different evidentiary limits. Hyaluronan enrichment did not select benefit from PEGPH20 in randomized HALO-301 (50). The vismodegib phase 2 study was not randomized and did not demonstrate improvement against historical expectations (41). Thus, these trials do not establish that all CAF-directed interventions are ineffective or that a particular adaptive mechanism caused failure.

We separate four evidence categories. E1 denotes preclinical mechanism demonstrated by perturbation in cells, organoids or animals. E2 denotes retrospective human association, including archived samples from a prospective trial when the biomarker analysis is retrospective. E3 denotes prospective biomarker or pharmacodynamic assessment, with prespecified sampling and assay evaluation. E4 denotes an interventional clinical study, annotated by phase, comparator and outcome. The categories can coexist and are not an automatic ladder to utility: an E4 trial can be negative, and exploratory biomarkers measured within it remain unvalidated for selection. The 2026 randomized paricalcitol run-in study supplies early interventional and serial-biopsy evidence, but its safety-led design does not establish survival benefit or a validated CAF-based selection rule (84). **Table 3** and **4** use these categories to separate mechanism from clinical maturity.

**Table 3:** CAF-related mechanisms of therapy resistance and candidate interventions.

Treatment setting	CAF-related mechanism	Evidence category and boundary	Candidate intervention	Key references
Gemcitabine and cytotoxic therapy	ECM deposition and CDH11-dependent fibrosis with immune suppression	E1. Mouse PDAC models.	CDH11 or matrix-signaling inhibition with chemotherapy	(87)
Chemotherapy after neoadjuvant exposure	IL6-family CAF-malignant interactions	E1 + E2. Human spatial association with ex vivo functional validation.	IL6/JAK/STAT-directed combination selected by pathway activity	(4)
Gemcitabine/nab-paclitaxel	Chemotherapy-induced PlGF/VEGF activates collagen-producing CAFs	E1 + E2. Experimental models and human association.	PlGF/VEGF blockade linked to PD-L1 targeting	(56)
Platinum	CAF IL8 induces UPK1A-AS1 and double-strand break repair	E1 + E2. Patient-derived CAF comparisons and experimental models.	IL8/NF-κB or repair-pathway inhibition	(88)
DNA-damaging chemotherapy	ECM-integrin-Src-FAK-SGK-NDRG1 supports R-loop processing	E1 + E2. Human cohort association and mechanistic models.	Integrin/FAK/SGK/NDRG1 pathway cotargeting	(69)
Radiotherapy plus checkpoint blockade	CCR2/CCR5-positive myeloid recruitment limits effector T-cell entry	E1 + E2. Mainly preclinical, with human correlative data.	CCR2/CCR5 inhibition with radiation and immune therapy	(89)
Immune checkpoint blockade	LRRC15-positive or senescent CAF programs suppress T cells	E1 + E2. Human association and mouse perturbation.	State-selective inhibition or senolysis with checkpoint therapy	(15–17)
Immunochemotherapy	CAF autophagy maintains desmoplasia and adaptive immune resistance	E1. Preclinical.	CAF-directed autophagy inhibition with chemoimmunotherapy	(76)
KRAS inhibition	Rapid TME remodeling and survival of classical epithelial states	E1. Mouse PDAC models. Epithelial resistance findings do not demonstrate CAF conversion.	KRAS inhibitor plus checkpoint or state-directed therapy	(58–60)

E1, preclinical mechanism; E2, retrospective human association; E3, prospective biomarker or pharmacodynamic assessment; E4, interventional clinical evidence. Categories describe the cited study and can coexist. Candidate interventions in this table are research hypotheses. None of these mechanism studies establishes a validated predictive clinical assay.

**Table 4:** Stromal therapeutic strategies organized by intent and clinical evidence.

Therapeutic intent and approach	Intended tissue effect	Combination or context	Evidence and clinical status	Required readout and limitation	References
Selective depletion Senescent-cell removal	Remove pathogenic senescent stromal cells	Checkpoint or cytotoxic therapy in models	E1; mouse efficacy and human descriptive support (E2).	Loss of the identified senescent CAF population; senolytics also affect other cells.	(16, 17)
Selective depletion FAP-directed approaches	Remove antigen-positive cells	Investigational CAF-directed strategies	Target rationale in the cited PDAC evidence; no efficacy conclusion is assigned to all FAP products.	Demonstrate which FAP-positive states were lost; preserve protective and normal populations.	(18)
Circuit interruption Hedgehog/vismodegib	Alter Hedgehog-dependent fibroblast programs	Gemcitabine/nab-paclitaxel	E1 + E4. Nonrandomized phase 2; no improvement against historical expectations.	Measure state composition; absence of a randomized comparator limits efficacy inference.	(32, 41)
Extracellular circuit/substrate Hyaluronan/PEGPH20	Reduce matrix pressure	Gemcitabine/nab-paclitaxel	E4. Randomized phase 3 HALO-301; no OS or PFS improvement in hyaluronan-high PDAC.	Perfusion and drug exposure; matrix digestion is not CAF depletion.	(50)
Circuit interruption PD-L1-directed PlGF/VEGF blockade	Reduce collagen-producing CAF activity	Chemotherapy	E1, with human correlative support (E2); no clinical benefit established in the cited study.	Pathway engagement and perfusion; multiple responding cell types.	(56)
Circuit interruption MEK plus STAT3 inhibition	Change inflammatory output and CAF composition	Checkpoint blockade	E1; experimental PDAC.	Immune access and state distribution; changes may include reprogramming.	(57)
Circuit interruption CAF autophagy inhibition	Reduce stromal support and adaptive immune resistance	Immunochemotherapy	E1; experimental PDAC.	Cell-resolved autophagy and immune response; systemic effects complicate attribution.	(76)
Indirect circuit remodeling KRASG12D inhibition	Alter epithelial signals and fibroblast/immune context	Checkpoint or state-directed therapy	E1 for the CAF mechanisms in the cited studies; not a summary of all KRAS drug development.	Track malignant and stromal states separately; primary target is in cancer cells.	(58–60)
Reprogramming VDR activation	Induce a less activated stromal program	Gemcitabine in mouse studies; gemcitabine/nab-paclitaxel in human trials	E1 + E3/E4. Randomized safety run-in (36 patients) with serial biopsies; separate single-arm phase 2 stopped at its interim efficacy gate.	Stromal/immune pharmacodynamics and hypercalcemia monitoring; survival benefit and selection assay unproven.	(84, 91, 93)
Reprogramming Retinoid/Am80	Induce Meflin and reduce crosslinking	Cytotoxic chemotherapy	E1; preclinical efficacy in the cited study.	Meflin induction plus matrix/function readouts; durability and human benefit unresolved.	(92)
Reprogramming Macropinocytosis inhibition	Change nutrient-stress adaptation and myCAF identity	Model-based combination studies	E1; mechanistic PDAC study.	State and viability measured together; a phenotypic shift is not cell depletion.	(23)

E1–E4 are defined in Section 6.2. E4 records clinical testing, not proven benefit, and E3 does not necessarily establish predictive utility. OS, overall survival; PFS, progression-free survival. Classification follows intended biological effect; a drug may affect more than one category. Clinical status describes the cited regimens, not every agent acting on the pathway.

### 6.3 Single-cell and spatial approaches to patient stratification

Single-cell and spatial methods can separate cancer-cell and CAF programs that are mixed in bulk tissue. Single-nucleus RNA sequencing (snRNA-seq) and digital spatial profiling identified multicellular communities in treated and untreated PDAC (3). These communities included classical, basal-like and treatment-enriched features. Other spatial studies mapped therapy-associated changes in ligand-receptor interactions and regional cell composition (4). Three-dimensional reconstruction and multimodal ECM imaging further show that two-dimensional sections can miss connected niches and matrix organization (85, 86).

For clinical use, the goal is not to reproduce a research atlas for every patient. A discovery dataset should yield a smaller assay using robust RNA or protein markers and spatial rules. Candidate outputs include the proportion of a harmful CAF state, its distance from tumor or T cells, and evidence of an active pathway. Repeated sampling is a challenge, but core biopsy, resection tissue, blood markers and imaging may provide complementary views.

Analytical reproducibility is equally important. Dissociation loses spatial information and can alter stress genes. snRNA-seq works with frozen tissue but samples nuclear RNA and may underrepresent some immune states. Spatial platforms differ in resolution and gene coverage. Classifiers trained on one platform require explicit transfer and validation before clinical use (20).

### 6.4 Barriers to clinical translation

Four barriers recur. First, markers overlap across CAF states and other mesenchymal cells. Second, CAF composition varies within and between lesions. Third, treatment changes the state being measured. Fourth, most causal evidence comes from mouse or simplified co-culture systems. Human tissue studies provide essential relevance but are often observational and limited by sample size.

Clinical development should address these barriers directly. Trials should collect baseline and on-treatment tissue where feasible, report stromal pharmacodynamics and prespecify how a CAF biomarker will guide analysis. Biomarkers should combine identity and function, for example LRRC15 with TGF-β activity and spatial T-cell exclusion, rather than use one marker alone. Preclinical testing should include patient-derived models with matched fibroblasts and immune components, together with genetically engineered models that preserve disease evolution.

The field also needs negative controls and transparent nomenclature. A proposed state should be compared with existing signatures, validated at the protein level and tested across cohorts. Claims of clinical prediction should be reserved for treatment interactions. These standards may slow the addition of new labels, but they increase the chance that a CAF measurement can guide care.

## 7 CAF-mediated therapy resistance

### 7.1 Chemotherapy resistance

CAFs reduce chemotherapy response through several routes. Matrix can alter perfusion and drug distribution. Cytokines activate survival pathways. Metabolites support stressed cancer cells. Direct matrix sensing can increase DNA repair. These mechanisms can coexist within one tumor, so decompression alone may not restore sensitivity.

Human and model data support specific pathways. Therapy-associated IL6-family signaling between CAFs and malignant cells conferred resistance in spatial and ex vivo studies (4). Chemotherapy-induced PlGF/VEGF signaling activated collagen-producing CAFs, and dual blockade improved drug effects in preclinical models (56). CDH11 promoted ECM deposition, immune suppression and gemcitabine resistance in mice (87). In patient-derived CAF comparisons and experimental models, CAF-derived IL8 induced a long noncoding RNA program that improved double-strand break repair and platinum resistance (88). CAF ECM also supported NDRG1-dependent R-loop processing and resistance to DNA-damaging chemotherapy (69).

Patient-specific models show that both magnitude and mechanism vary. In matched organoid-CAF co-cultures, CAFs increased proliferation and reduced chemotherapy-induced death, but responses differed between pairs (83). Variation between pairs supports functional testing, yet organoids cannot predict clinical benefit on their own. Integration with genomic, spatial and pharmacokinetic data will be needed.

### 7.2 Radiotherapy resistance

Radiotherapy damages cancer cells but also alters stromal and immune compartments. Treated human tumors contain distinct multicellular programs, and combined chemotherapy/radiotherapy changes fibroblast-malignant interactions (3, 4). Radiation can increase antigen release and T-cell priming while simultaneously recruiting suppressive myeloid cells.

In a PDAC model, dual CCR2/CCR5 inhibition permitted radiation-induced effector T-cell infiltration and improved combination immunotherapy (89). The study included human correlative observations but remained primarily preclinical. Future studies should separate fibroblast-intrinsic radioresponse from secondary effects of tissue injury, vascular damage and myeloid recruitment.

### 7.3 Immunotherapy resistance

CAF-mediated immunotherapy resistance is generated by immune exclusion, suppressive cytokines, antigen-presentation context and myeloid feedback. In mouse perturbation studies, LRRC15-positive and senescent CAF programs restrict antitumor immunity (15–17). HIF2-dependent fibroblast signaling supports suppressive macrophages and Tregs (73). NetG1-positive CAFs limit NK-cell killing (53). These mechanisms are partly nonredundant, which helps explain why blocking one checkpoint is insufficient.

Combination studies aim to dismantle the dominant circuit. TLR9 agonism, MEK/STAT3 inhibition and CAF-autophagy targeting have each improved checkpoint-based therapy in preclinical models (57, 75, 76). KRASG12D inhibition increased CD8-positive T cells and remodeled CAFs; checkpoint blockade further deepened responses in advanced mouse models (58). The evidence supports mechanism-based combinations, not indiscriminate addition of a stromal drug to immunotherapy.

Patient selection should identify both a targetable CAF function and a potentially recoverable immune response. A tumor with severe T-cell absence may need priming or trafficking support before checkpoint blockade. A tumor with T cells trapped outside malignant glands may benefit from interruption of a CAF-myeloid circuit. The required biomarker is therefore spatial and mechanistic.

### 7.4 Therapy-induced plasticity and recurrence

Acquired resistance may involve both stress-induced adaptation and selection of pre-existing states. Cytotoxic injury changes fibroblast signaling and extracellular resources; surviving CAFs can then maintain cytokine, matrix and metabolic support for residual cancer cells (4, 5, 8). Model perturbations show that nutrient uptake and signaling inhibition can shift CAF programs (23, 57). By contrast, the classical epithelial reservoir selected during KRAS inhibition is a malignant-cell finding and must not be presented as direct evidence of CAF lineage conversion (58–60).

A testable resistance sequence is exposure, altered stress-response output, a persistent supportive niche and reduced sensitivity to subsequent therapy. Demonstrating this sequence requires temporal ordering plus perturbation of the proposed CAF mediator. Longitudinal human association alone cannot establish the sequence. Recovery after stress withdrawal would support reversible adaptation; persistence after withdrawal would motivate tests of epigenetic stabilization, senescence or selection. The framework does not assume that all stress responses are harmful or reversible.

This plasticity favors adaptive treatment designs. Baseline state should guide the initial combination, while early on-treatment sampling should determine whether the intended shift occurred or a resistant niche emerged. Forcing a harmful state into a more tractable one may be more useful than trying to maintain a fixed composition. **Table 3** links major resistance mechanisms to candidate interventions and their evidence boundaries.

## 8 Therapeutic strategies targeting CAFs and the stroma

### 8.1 Selective depletion of pathogenic states

The therapeutic intent should be stated before the target. Selective depletion removes cells that causally sustain a harmful state. Circuit interruption blocks a defined stromal interaction while retaining the cells. Reprogramming or normalization changes cell function or tissue organization toward a less tumor-supportive condition. These intents may overlap pharmacologically, but require different pharmacodynamic endpoints. Matrix digestion is not CAF depletion, and a lower marker signal alone cannot distinguish cell loss from altered expression.

CAF depletion is conceptually simple but biologically risky. FAP is expressed by many activated fibroblasts and has been used for antibody, drug-conjugate, radioligand and cellular therapy strategies. Yet FAP-positive cells are heterogeneous, and recent work identified a tumor-restraining interferon-response population within this compartment (18). Depletion may also affect normal tissue repair or other FAP-positive mesenchymal cells.

Mouse genetic studies warn against broad depletion. Removal of selected myofibroblast-associated compartments or their collagen accelerated PDAC (11, 33), and Meflin-positive cell ablation increased tumor aggressiveness (70). In contrast, eliminating senescent stromal cells improved antitumor responses in mouse PDAC (16, 17). Senolysis therefore belongs under selective depletion, subject to evidence that the targeted senescent cells are pathogenic and that the intervention spares essential non-CAF populations.

FAP-directed chimeric antigen receptor approaches and other depletion methods should include state-resolved pharmacodynamics. A reduced FAP signal is insufficient. Studies should measure which CAF programs were removed, whether immune access improved and whether compensatory inflammatory or senescent states expanded. In PDAC, depletion is most defensible when a restricted surface marker is tied to a causal function and treatment can be stopped or controlled.

### 8.2 Interruption of CAF-mediated circuits

Circuit interruption aims to reduce a measurable output, such as cytokine-mediated survival, immune exclusion or matrix-dependent signaling, without requiring fibroblast loss. Candidate pathways include IL-1/JAK/STAT, OSM-OSMR, CXCL1-TNF, TGF-β and integrin-FAK signaling (4, 22, 34, 40, 69). Reduced ligand activity, receptor signaling or the relevant cellular interaction is the appropriate endpoint. Pathway use by malignant and immune cells limits claims of CAF specificity.

TGF-β blockade may reduce LRRC15-positive myofibroblastic suppression, but it can also remove restraining myCAF functions or alter T-cell and epithelial biology (15, 22, 33). JAK/STAT or IL6-family inhibition may suppress inflammatory CAF support, especially after treatment-induced pathway activation (4). FAK and integrin pathways link ECM sensing to survival, DNA repair and KRAS-inhibitor response (69, 90). These targets are most likely to succeed when the downstream functional state is measured.

Hedgehog inhibition provides a clinical lesson. It changes fibroblast composition rather than simply removing stroma (32), and vismodegib did not improve chemotherapy outcome in the tested phase 2 setting (41). A future signal-directed trial should specify the predicted state transition, confirm it early and combine treatment with an agent that exploits the new state.

### 8.3 Functional reprogramming and state normalization

Reprogramming seeks to restore quiescent or tumor-restraining fibroblast functions. Vitamin D receptor activation moved pancreatic stellate cells toward a more quiescent program, reduced inflammatory and fibrotic features, improved intratumoral gemcitabine exposure and prolonged survival in mice (91). A synthetic retinoid, Am80, induced Meflin, reduced tissue stiffening through lysyl oxidase regulation and improved chemotherapy sensitivity in preclinical PDAC (92).

Clinical evidence now extends beyond the original mouse proof of concept. Perez et al. randomized 36 patients with metastatic pancreatic cancer to chemotherapy with placebo, intravenous paricalcitol or oral paricalcitol in a safety-led run-in trial. Serial biopsies showed changes in fibroblast and CD8 T-cell features, while oral treatment required dose reductions for hypercalcemia (84). These findings support pharmacodynamic feasibility rather than definitive efficacy. In a separate single-arm phase 2 study, oral paricalcitol plus chemotherapy failed to meet the prespecified threshold to continue enrollment (93). Differences in design and regimen preclude treating the studies as interchangeable; durable clinical benefit from VDR-mediated stromal reprogramming remains unproven.

For retinoid and metabolic approaches, evidence remains principally preclinical in the cited studies (23, 92). Functional normalization should be demonstrated by a favorable change in matrix organization, perfusion, immune access or treatment sensitivity, alongside preserved fibroblast viability where reprogramming is intended. Reduced α-SMA alone is not sufficient. Washout studies should assess persistence, and monitoring should detect compensatory inflammatory or senescent programs.

### 8.4 Rational combination strategies

CAF therapy is unlikely to be effective as a universal single agent because CAF functions protect cancer through several mechanisms. Combinations should be built from an evidence chain: identify the dominant stromal function, block it, and pair the intervention with a treatment that becomes effective after the block.

Examples illustrate this logic. PD-L1-directed PlGF/VEGF blockade reduced collagen-producing CAF activity and improved chemotherapy response in models (56). Radiation plus CCR2/CCR5 inhibition relieved a myeloid trafficking barrier and permitted effector T-cell entry (89). TLR9 agonism provided immune priming before checkpoint blockade (75). KRASG12D inhibition remodeled CAFs and increased CD8-positive T-cell killing, creating a rationale for checkpoint combination (58). These combinations target connected steps rather than adding drugs with unrelated activity.

Combination trials should specify the therapeutic intent, the clinical evidence category and the intended early tissue effect. The negative PEGPH20 result and the nonrandomized vismodegib findings constrain those regimens, while early paricalcitol trials show why pharmacodynamic activity must be separated from efficacy (41, 50, 84, 93). **Table 4** groups representative approaches by depletion, circuit interruption and reprogramming, retaining matrix-directed therapy as a distinct intervention on the extracellular substrate.

## 9 Future directions

### 9.1 Moving beyond fixed subtypes

A useful next step is an explicit research workflow that integrates subtype anchors with functional state and evidence maturity (**Figure 3**). Recent frameworks already combine molecular phenotypes and spatial archetypes (94). Building on that work, we propose a PDAC-focused decision sequence that asks whether a measured program is reproducible, clinically associated, causally involved and sufficiently validated to inform an intervention. Its contribution is operational integration rather than a claim to have discovered a new universal CAF taxonomy.

**Figure 3:**
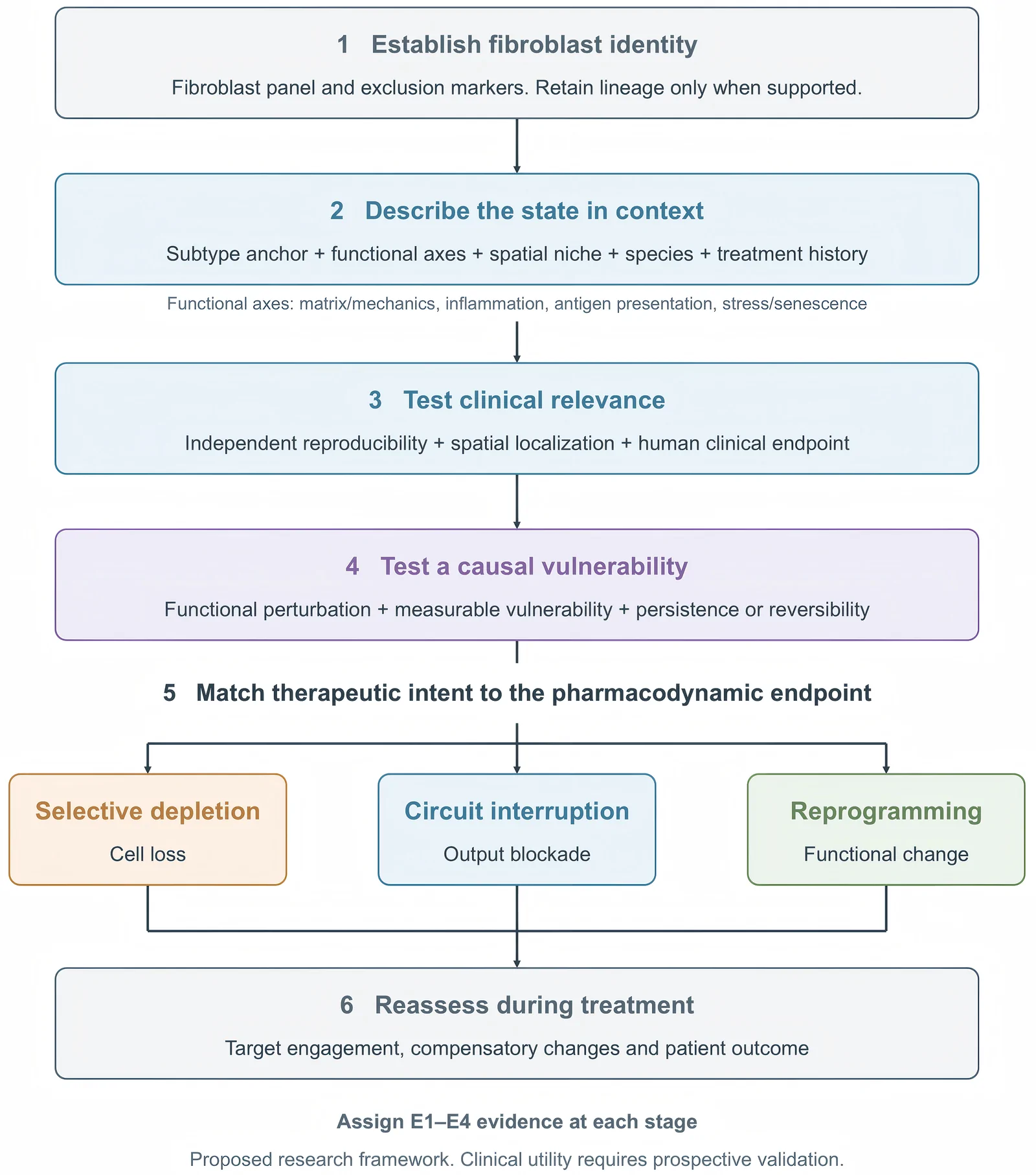
Proposed workflow for integrating CAF subtype, functional state and clinical evidence. The sequence combines fibroblast identity with functional axes, spatial context, treatment history and species-specific evidence. An expression-defined program progresses to a clinically relevant candidate only when reproducibility and human endpoint association are established. Functional perturbation and an intervention-accessible vulnerability support trial prioritization; validated actionability additionally requires prospective clinical utility. The choice among depletion, circuit interruption and reprogramming determines the pharmacodynamic endpoint. On-treatment reassessment tests target engagement and compensatory changes. The workflow is an author-proposed research framework, informed by existing molecular and spatial classifications (20, 21, 39, 94), and is not a validated scoring system or clinical prescribing algorithm. E1, preclinical mechanism; E2, retrospective human association; E3, prospective biomarker or pharmacodynamic assessment; E4, interventional clinical evidence.

First, establish fibroblast identity with an exclusion panel and retain lineage information only when directly supported. Second, record canonical subtype resemblance together with matrix/mechanical, inflammatory, antigen-presentation and stress/senescence outputs. Third, annotate spatial niche, species, lesion site and treatment history. Fourth, evaluate clinical relevance and transition evidence using Section 2.4 and assign E1-E4 evidence categories. A human MHC II-high fibroblast should not be called mesothelial-derived solely from its expression profile. Origin may remain unknown while its functional and spatial features are measurable.

Fifth, test whether perturbing the candidate state or circuit changes the relevant tumor or immune function in appropriate models. If clinical association alone is available, retain the state as a candidate prognostic biomarker; if causal dependency and a measurable vulnerability are supported, prioritize it for a biomarker-stratified trial. Choose depletion when a pathogenic population can be selectively removed, circuit interruption when its output is separable from cell identity, or reprogramming when beneficial function can be induced without loss of protective stroma. Sixth, repeat state and tissue-function measurements during treatment. Failure of target engagement or emergence of an adverse compensatory niche should trigger reassessment within a prespecified study protocol. This workflow is a proposal for prospective testing, not a validated clinical treatment algorithm.

### 9.2 Translating single-cell and spatial data into clinical assays

Research atlases should be converted into assays that work on routine tissue. Multiplex immunohistochemistry, targeted RNA panels and spatial proteomics can measure a limited set of states and neighborhoods. The assay should include tumor-cell state, because CAF and epithelial programs independently predict outcome and can modify one another (3, 68, 79). It should also include treatment history.

Validation must cross platforms and centers. A classifier derived by single-cell RNA sequencing (scRNA-seq) should be tested in frozen and formalin-fixed tissue, with reproducible thresholds and blinded outcome analysis. Three-dimensional methods show that tissue architecture is richer than one section, but clinical deployment may use carefully selected two-dimensional features that predict the larger structure (85, 86). The aim is a stable decision tool, not maximal resolution.

### 9.3 Precision stromal therapy

Precision stromal therapy requires matching a mechanism to a patient and a time point. An LRRC15-high, TGF-β-active and T-cell-excluded niche is a hypothesis for circuit-directed study, whereas a pathogenic senescent population may motivate selective depletion. These examples specify different tests and pharmacodynamic endpoints; they are not current clinical prescribing rules.

Patient-derived organoid-CAF systems can support this matching by testing pair-specific drug effects (83). Their predictive use will improve if immune and vascular components are added and if results are compared with patient response. Spatial profiling before and during therapy can reveal whether the targeted niche changed. Circulating or imaging markers may reduce the need for repeated invasive biopsy.

Negative selection should also be investigated. Human associations involving Meflin-positive, interferon-response or complement-associated programs, together with tumor-restraining effects in experimental systems, justify testing whether these functions should be preserved (18, 19, 70). They do not yet identify patients who will be harmed by a particular stromal drug.

### 9.4 From elimination to controlled remodeling

Controlled remodeling encompasses the three therapeutic intents in Section 8. Selective depletion requires a cell-loss endpoint, circuit interruption a pathway or interaction endpoint, and reprogramming a favorable functional change with evidence of cell persistence. A shared clinical requirement is demonstration of benefit beyond target engagement. Current VDR studies illustrate the gap between stromal modulation and established therapeutic efficacy (84, 93).

Controlled remodeling should be assessed longitudinally. CAF plasticity means that a successful initial change can be followed by compensation. Adaptive trial designs could use early tissue or imaging results to add a second agent only when a resistant state appears. Although more complex than conventional development, this approach matches the biology observed in treatment-associated spatial studies (4, 5).

## 10 Conclusions

CAFs regulate PDAC tissue structure, metabolism, immunity and therapy response. The canonical myCAF, iCAF and apCAF framework made this heterogeneity experimentally tractable. Subsequent studies have shown that these groups contain overlapping programs and that cells can change phenotype in response to cytokines, mechanics, metabolic stress and treatment. LRRC15-positive, senescent, interferon-response and complement-secreting populations add distinct functions. They belong in a common functional framework rather than as isolated, permanent types.

The main clinical lesson is that stromal abundance does not identify stromal dependence. Species, matrix source, local cellular interactions and treatment exposure alter the meaning of a CAF measurement. Negative stromal trials constrain specific interventions, while early pharmacodynamic studies support further testing without establishing clinical utility.

A clinically relevant CAF assessment should combine reproducible identity and function with spatial context and human outcome evidence. Actionability additionally requires a causal vulnerability and prospective demonstration that a state-informed decision improves treatment selection or outcome. Selective depletion, circuit interruption and functional reprogramming should be evaluated with distinct pharmacodynamic endpoints and longitudinal monitoring.

The clinically relevant questions are which fibroblast function dominates in a particular niche, whether that function is stable or reversible and how changing it alters the tumor ecosystem. Validated markers and prospective clinical studies will determine whether CAF biology becomes a practical component of precision therapy for PDAC.
